# iTRAQ-based quantitative proteomic analysis of alterations in the intestine of Hu sheep under weaning stress

**DOI:** 10.1371/journal.pone.0200680

**Published:** 2018-07-19

**Authors:** Kai Cui, Bo Wang, Naifeng Zhang, Yan Tu, Tao Ma, Qiyu Diao

**Affiliations:** Feed Research Institute, Chinese Academy of Agricultural Sciences, Beijing, China; Hospital Universitari i Politecnic La Fe, SPAIN

## Abstract

When Lambs are weaned off ewe’s milk, metabolic, structural, and functional changes often occur in the small intestine. Because information on the effects of weaning stress on the proteome of the intestine is limited, an animal model was established with eight pairs of twin lambs divided into artificially reared and ewe-reared groups, which was followed by proteome analysis using iTRAQ technology. Changes occurred in the morphology of the intestine and 5,338 proteins in three biological replicates with less than a 1.2% false discovery rate were identified and quantified. Among them, a subset of 389 proteins were screened as significantly up- (143) and down-regulated (246) in artificially reared compared with ewe-reared. According to Gene Ontology and Kyoto Encyclopedia of Genes and Genomes pathway enrichment analysis, the differentially expressed proteins that were strongly down-regulated were enriched in immune system processes, biological adhesion, and metabolic processes. The up-regulated proteins were enriched in gene expression, cellular biosynthetic processes, ribosome and RNA binding in response to weaning stress. A series of proteins associated with intestine morphology and immune function were identified, and levels of the mRNAs encoding these proteins were analyzed by real-time quantitative reverse transcription PCR. The results of this study increased our understanding of the response of lambs weaned off ewe’s milk and helped to determine the mechanisms underlying weaning stress.

## Introduction

A current trend in large-scale livestock operations is to wean animals at a younger age to increase dam productivity [1, 2]. Weaning is one of the most stressful events in the life of a neonate, which is characterized by low feed intake, weight loss, and increased mortality [3]. At weaning, neonates are exposed to many stressors, such as the breakdown of the mother-young bond, the end of lactational immunity, the new interaction with other lambs, the replacement of milk by solid food and a change in their environment and gut microbiota [4–7]. After mammalian neonates are weaned from their mothers, tremendous changes occur in intestinal structure and function [8, 9].

The small intestine is the primary organ involved in the digestion, absorption, and metabolism of dietary nutrients, including proteins and amino acids [10]. Moreover, the gastrointestinal system has multiple functions in secreting digestive enzymes, mucin, immunoglobulins, and various other components, in addition to providing a defensive barrier against diet derived pathogens, carcinogens and oxidants [11–13]. With the sudden change of feeding regime after weaning, morphological and histological changes occur in the small intestine that are critical for the immature digestive system. Post-weaning syndrome, manifested as anorexia, intestinal atrophy, diarrhea, and growth retardation in mammalian neonates (including human infants), is a major problem in animal production and public health, particularly in developing countries [14, 15]. Therefore, methods must be developed to minimize behavioral and physiological responses to weaning.

The Chinese Hu sheep is an important indigenous breed widely raised in the Taihu Lake area of China. This sheep breed is known for its beautiful lambskin, early sexual maturity, and high fecundity (200–250%), and the sheep was listed as one of the 78 nationally protected domestic animals by the Chinese government in 2000 [16, 17]. In sheep production, weaning of lambs can be very stressful for both the dam and the offspring. The focus of previous studies was on post-weaning management strategies to reduce weaning stress and improve the welfare and the productive performance of farm animals. However, little is known about the molecular mechanisms of the intestinal response to diet change and weaning. Wang [10] assessed the effects of dietary acidification with sorbic acid on gene expression during weaning in pig (Sus scrofa) ileums with microarray technology and bioinformatics analyses.

Recently, the use of proteomics, i.e., the study of the proteome (or expressed proteins) under specific conditions, has led to much greater insight into the metabolic mechanisms of a vast array of physiological functions [18]. To better understand the challenges and mechanisms of the intestines associated with weaning and to help producers develop other management techniques that reduce weaning stress, the relative or absolute expression of proteins during the weaning process must be determined. Isobaric tags for relative and absolute quantification (iTRAQ) are a powerful proteomics method to quantify relative protein levels. Science twins reduce influence of genetic effects, in this study, twin lambs of Hu sheep were used and differentially expressed proteins were identified by iTRAQ. To increase our understanding of genetic and molecular mechanisms of the weaning process, the possible roles of these proteins are discussed in relation to intestine function.

## Materials and methods

### Ethics statement

This research was conducted at the Linqing Runlin Animal Husbandry Co., LTD, in Shandong, China. All experiments were conducted according to the Regulations for the Administration of Affairs Concerning Experimental Animals published by the Ministry of Science and Technology, China, in 2004. The Chinese Academy of Agricultural Sciences Animal Ethics Committee approved all experiments, and humane animal care and handling procedures were followed throughout the experiment.

### Animals and diets

Eight pairs of twin neonatal ram Hu lambs born on the same day with an average body weight of 2.89±0.36 kg were randomly divided into two groups. Eight lambs (one lamb from each pair of twins) separated from the ewes were artificially reared (AR) with milk replacer which acquired from Precision Animal Nutrition Research Center (Beijing, China) after the lambs had been sucking colostrum for three days. These lambs were fed four times a day, and the feed amount of milk replacer was adjusted in direct accordance with 2% of the lamb’s body weight. The other 8 lambs were ewe-reared (ER) during the entire experiment, which continued for 15days.

**Fig 1:**
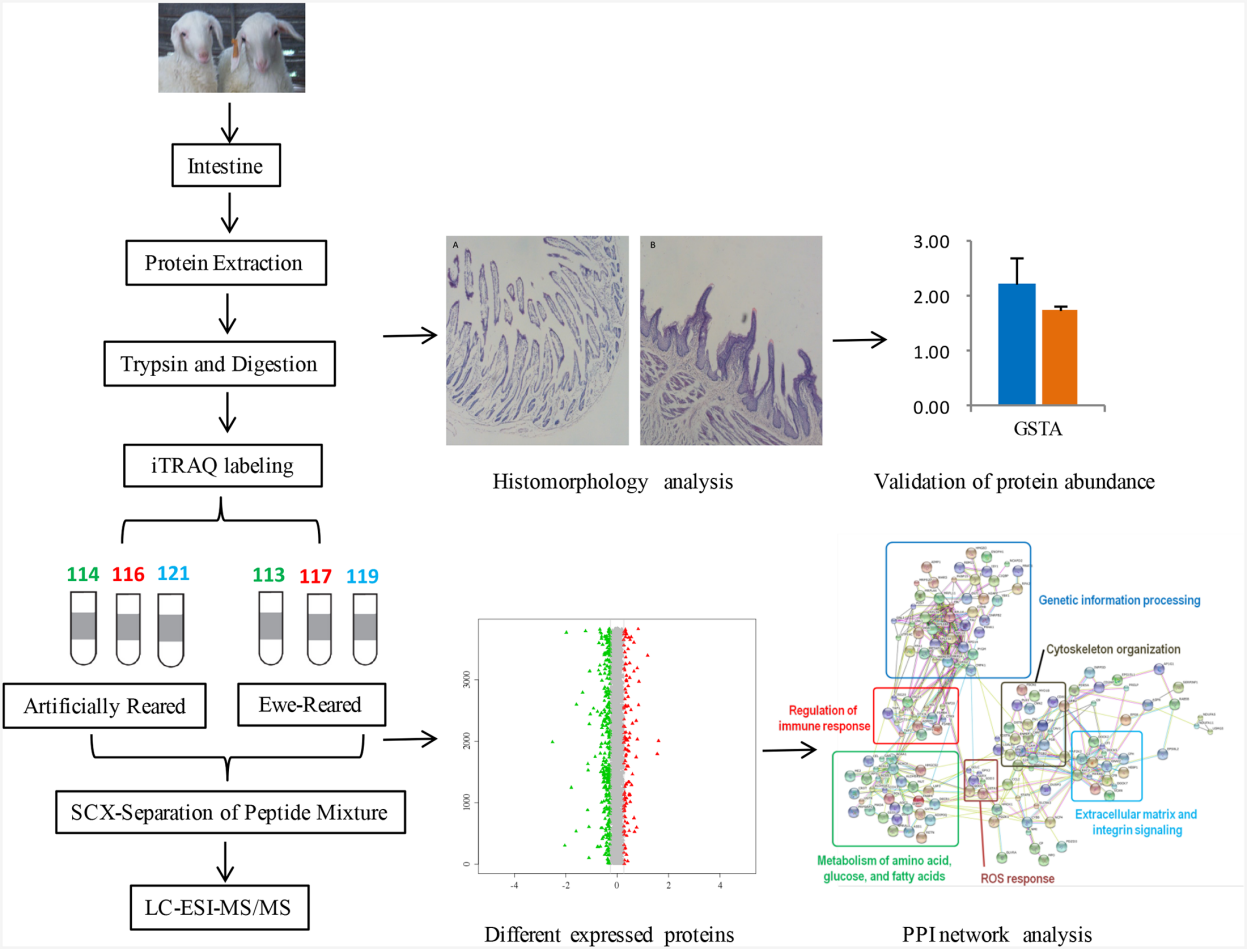
Schematic representation of the experimental design to compare weaning-stressed intestines of artificially reared lambs with ewe-reared lambs (control) using the iTRAQ method.

On the 15^th^ day, three pairs of twins were weighed and then slaughtered with a compressed air pistol to cause a cerebral concussion, followed by exsanguination by cutting the jugular and carotid veins. After slaughter, samples of the jejunum tissue were quickly harvested and frozen in liquid nitrogen for total protein extraction (Fig 1).

### Histomorphology analysis

Intestinal tissues from ER and AR lambs were fixed in 4% paraformaldehyde (Sigma-Aldrich, Wisconsin, America). The samples were dehydrated with an ethanol and toluene (Beijing Chemical Works) series and embedded in paraffin (Leica, Wetzlar, Germany). Serial sections (6 μm thickness) were mounted on gelatin-coated glass slides and stained with hematoxylin and eosin (H&E).

### Protein isolation and iTRAQ labeling

The jejunum samples were ground into powder in liquid nitrogen and then extracted with lysis buffer (7 M urea, 2 M thiourea, 4% CHAPS, 40 mM Tris-HCl, pH 8.5). The suspension was sonicated at 200 W for 15 min and then centrifuged at 4°C and 30,000 g for 15 min. The supernatant was transferred to another tube and 5× volume of chilled acetone containing 10% (v/v) TCA and incubated at -20°C overnight to precipitate proteins. The supernatant was discarded after centrifugation at 4°C and 30,000 g for 15 min, and the precipitate was washed with chilled acetone three times. The pellet was air-dried for 5 min, dissolved in 500 μl of 0.5 M TEAB (Applied Biosystems, Ohio, America), and sonicated at 200 W for 15 min. Finally, the samples were centrifuged at 4°C and 30 000 g for 15 min, and the supernatant was quantified for protein content using a BCA Protein Assay Kit from Biyotime (Suzhou, China) with BSA as a standard. The proteins in the supernatant were stored at -80°C for further analysis.

The total protein (100 μg) from each sample was digested with trypsin with a ratio of protein:trypsin of 30:1 at 37°C for 16 h. After trypsin digestion, peptides were dried by vacuum centrifugation, reconstituted in 0.5 M TEAB and processed according to the manufacturer’s protocol for an 8-plex iTRAQ kit (Applied Biosystems, Ohio, America). The ER replicates were labeled with iTRAQ tags 113, 117, and 119, and the AR replicates were labeled with tags 114, 116, and 121. The peptides were labeled with isobaric tags and incubated at room temperature for 2 h. The labeled peptide mixtures were then pooled and dried by vacuum centrifugation.

### Strong cationic-exchange chromatography separation

SCX chromatography was performed with an LC-20AB HPLC Pump system (Shimadzu, Kyoto, Japan). The iTRAQ-labeled peptide mixtures were reconstituted with 4 ml of buffer A (25 mM NaH_2_PO_4_ in 25% ACN, pH 2.7) and loaded onto a 4.6×250 mm Ultremex SCX column containing 5-μm particles (Phenomenex, Torrance, America). The peptides were eluted at a flow rate of 1 ml/min with a gradient of 100% buffer A for 10 min, 5–60% buffer B (25 mM NaH_2_PO_4_, 1 M KCl in 25% ACN, pH 2.7) for 27 min, and 60–100% buffer B for 1 min. The system was then maintained at 100% buffer B for 1 min before equilibrating with buffer A for 10 min before the next injection. Elution was monitored by measuring the absorbance at 214 nm, and fractions were collected every 1 min. The eluted peptides were pooled into 20 fractions, desalted with a Strata X C18 column (Phenomenex, Torrance, America) and vacuum-dried.

### LC-ESI-MS/MS analysis based on Triple TOF 5600

Each fraction was resuspended in buffer A (5% ACN, 0.1%FA) and centrifuged at 20000 g for 10 min; the final peptide concentration was approximately 0.5 μg/μl on average. Then, 10 μl of the supernatant was loaded onto an LC-20AD nano HPLC (Shimadzu, Kyoto, Japan) by an auto sampler onto a 2 cm C18 trap column. Next, the peptides were eluted onto a 10 cm analytical C18 column (inner diameter 75 μm) packed in-house. The samples were loaded at 8 μl/min for 4 min. Then, the 35 min gradient was run at 300 nl/min starting from 2 to 35% B (95%ACN, 0.1%FA), followed by a 5 min linear gradient to 60% and a 2 min linear gradient to 80%. The gradient was then maintained at 80% B for 4 min before finally returning to 5% B in 1 min.

Data acquisition was performed with a Triple TOF 5600 System (AB SCIEX, Concord, ON) fit with a Nanospray III source (AB SCIEX, Concord, ON) and a pulled quartz tip emitter (New Objectives, Woburn, MA). Data were acquired using an ion spray voltage of 2.5 kV, a curtain gas of 30 psi, a nebulizer gas of 15 psi, and an interface heater temperature of 150°C. The MS was operated with an RP of greater than or equal to 30,000 FWHM for TOF MS scans. For IDA, survey scans were acquired in 250 ms and up to 30 product ion scans were collected, exceeding a threshold of 120 counts per second (counts/s) with a 2+ to 5+ charge-state. Total cycle time was fixed to 3.3 s. The Q2 transmission window was 100 Da for 100%. Four time bins were summed for each scan at a pulser frequency value of 11 kHz by monitoring the 40 GHz multichannel TDC detector with a four-anode channel detect ion. A sweeping collision energy setting of 35±5 eV coupled with iTRAQ adjusted rolling collision energy was applied to all precursor ions for collision-induced dissociation. Dynamic exclusion was set for 1/2 of the peak width (15 s), and then the precursor was refreshed off the exclusion list.

### Proteomic data analysis

Raw data files were processed in Proteome Discoverer 1.2 (PD 1.2, Thermo), and protein identification was performed using the Mascot search engine (version 2.3.02; Matrix Science) against the *Ovis aries* database containing 26,297 sequences (http://www.ncbi.nlm.nih.gov/protein/?term=txid9940[Organism:exp]). For protein identification, the following options were used: type of search = MS/MS ion search, enzyme = trypsin, fragment mass tolerance = 0.1 Da, variable modifications = Gln->pyro-Glu (N-term Q), oxidation (M), iTRAQ8plex (Y), peptide mass tolerance = 0.05 Da, max. missed cleavages = 1, fixed modifications = carbamidomethyl (C), iTRAQ4plex (N-term), and iTRAQ4plex (K). To reduce the probability of false peptide identification, only peptides at the 95% confidence interval by a Mascot probability analysis greater than “identity” were counted as identified. For each confident identification, the protein included at least one unique peptide. Proteins with 1.2-fold or more change between successive comparisons and a *p*-value of statistical evaluation less than 0.05 were determined as differentially expressed proteins.

Functional annotations of the proteins were conducted using the Blast2GO program against the non-redundant protein database (www.blast2go.com/). The Clusters of Orthologous Groups of proteins database (COG; http://www.ncbi.nlm.nih.gov/COG/) was used to classify and group the identified proteins. The Database for Annotation, Visualization and Integrated Discovery (DAVID v6.7; http://david.abcc.ncifcrf.gov) was used to annotate biological themes (gene ontology, GO). The Kyoto Encyclopedia of Genes and Genomes (KEGG; http://www.genome.jp/kegg/) was used to determine the associated pathways. Additionally, the differentially expressed proteins were sent to the Search Tool for the Retrieval of Interacting Genes/Proteins (STRING; http://string.embl.de/) to build functional protein association networks, which were used for comparisons. Phenotype annotations of differentially expressed proteins (DEPs) were analyzed based on the Mouse Genome Informatics (MGI; http://www.informatics.jax.org/phenotypes.shtml) database.

### Quantitative real-time PCR analysis of gene expression

In this research, several genes were selected for quantitative real time RT-PCR (qRT-PCR) analysis. The primers were designed with primer 5.0 and synthesized by Tsingke Biotech Co., Ltd. The genes and their primer sequences used for qRT-PCR analysis are listed in S1 Table. RNA extraction, cDNA synthesis and qRT-PCR were conducted according to the method described previously. The glyceraldehyde-3-phosphate dehydrogenase (GAPDH) gene was used as an internal control to normalize the expression data. Each qRT-PCR experiment was repeated three times. The relative expression of genes was calculated using the 2^-ΔΔCt^ method and the standard deviation was calculated based on three biological replicates.

## Results

### Effect of weaning stress on morphology of the intestines

**Fig 2:**
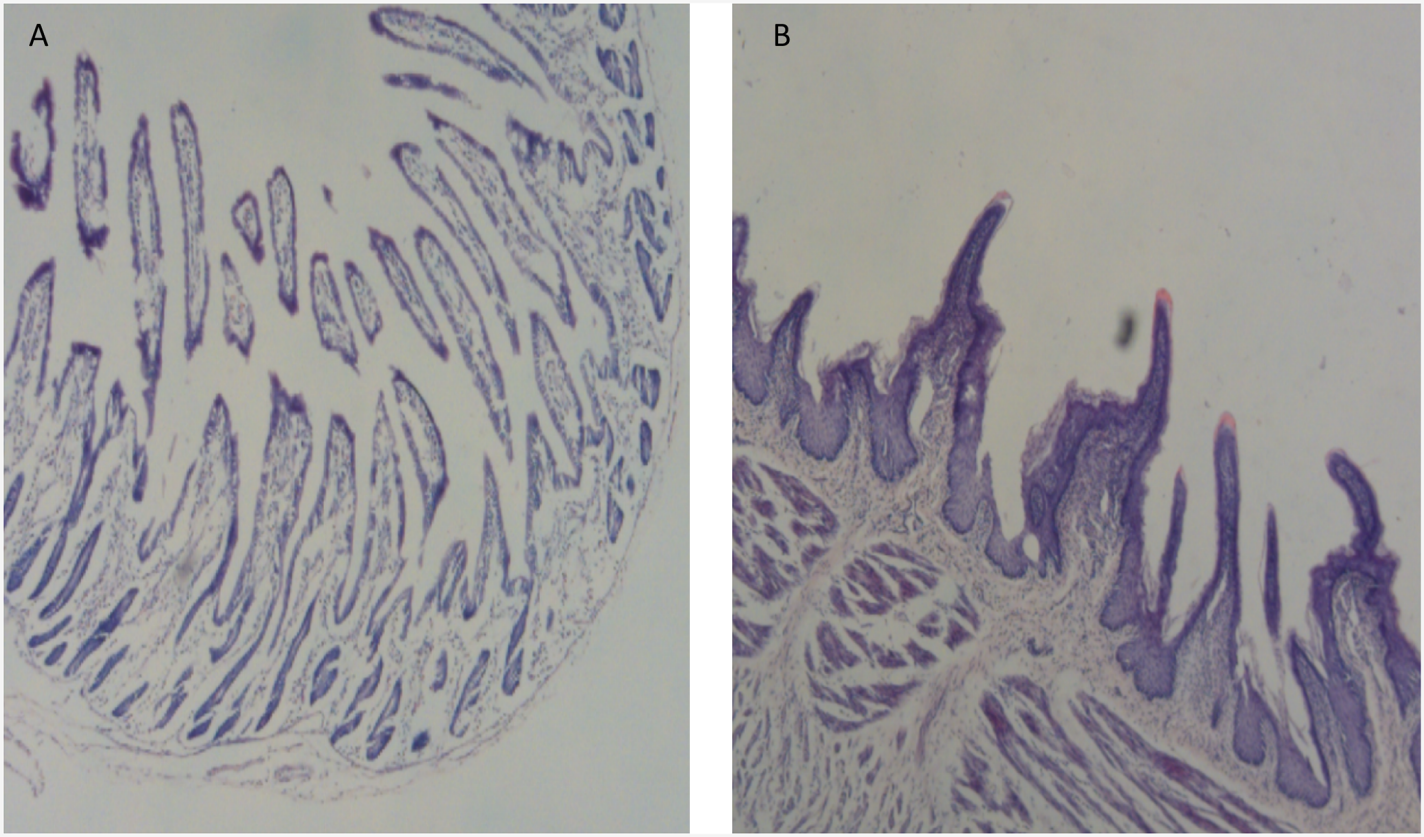
Phenotypic comparison of the intestines from ewe-reared and artificially reared lambs.

Histological sectioning determined the different morphological changes in the intestine between the two groups. Compared with the ER group, the weaned lambs of the AR group showed structural changes in the small intestine, including shortened villi (villous atrophy) and increased crypt depth (crypt elongation; Fig 2).

### Proteomics characterization

After the Mascot program search, a total of 76,038 high scoring unique spectra of 85,247 mass spectra that matched special peptides were obtained (S3 Fig). We identified 28,123 unique peptides from 29,761 peptides that corresponded to 5,338 proteins in the three different biological replicates with less than 1.2% FDR in each analysis (S1 Fig). All proteins identified by LC-ESI-MS/MS and their peptides are provided in Supporting Information S2 Table. Of these proteins, 69.5% were identified with at least two peptides, which permitted the abundance to be quantified (S3 Fig). The distribution of the protein sequence coverage is shown in S3 Fig.

To facilitate a deep global analysis of protein expression, the detected proteins were classified into 25 clusters of COG categories. The percentages of proteins involved in general function prediction only, posttranslational modification, protein turnover, and chaperones were dominant, indicating that posttranscriptional regulation played an important role in the intestine during weaning stress (S2 Fig).

### Functional annotation analysis of proteome differences

To further understand the differentially expressed proteins during the weaning process, DEPs were identified by the relatively relaxed criteria of fold change (>1.2-fold) and *p*-value (<0.05) between AR and ER groups. Finally, 389 differentially expressed proteins (7.29%) were identified by iTRAQ analysis, with 143 (2.68%) up-regulated and 246 (4.61%) were down-regulated under weaning stress conditions (S3 Table).

**Fig 3:**
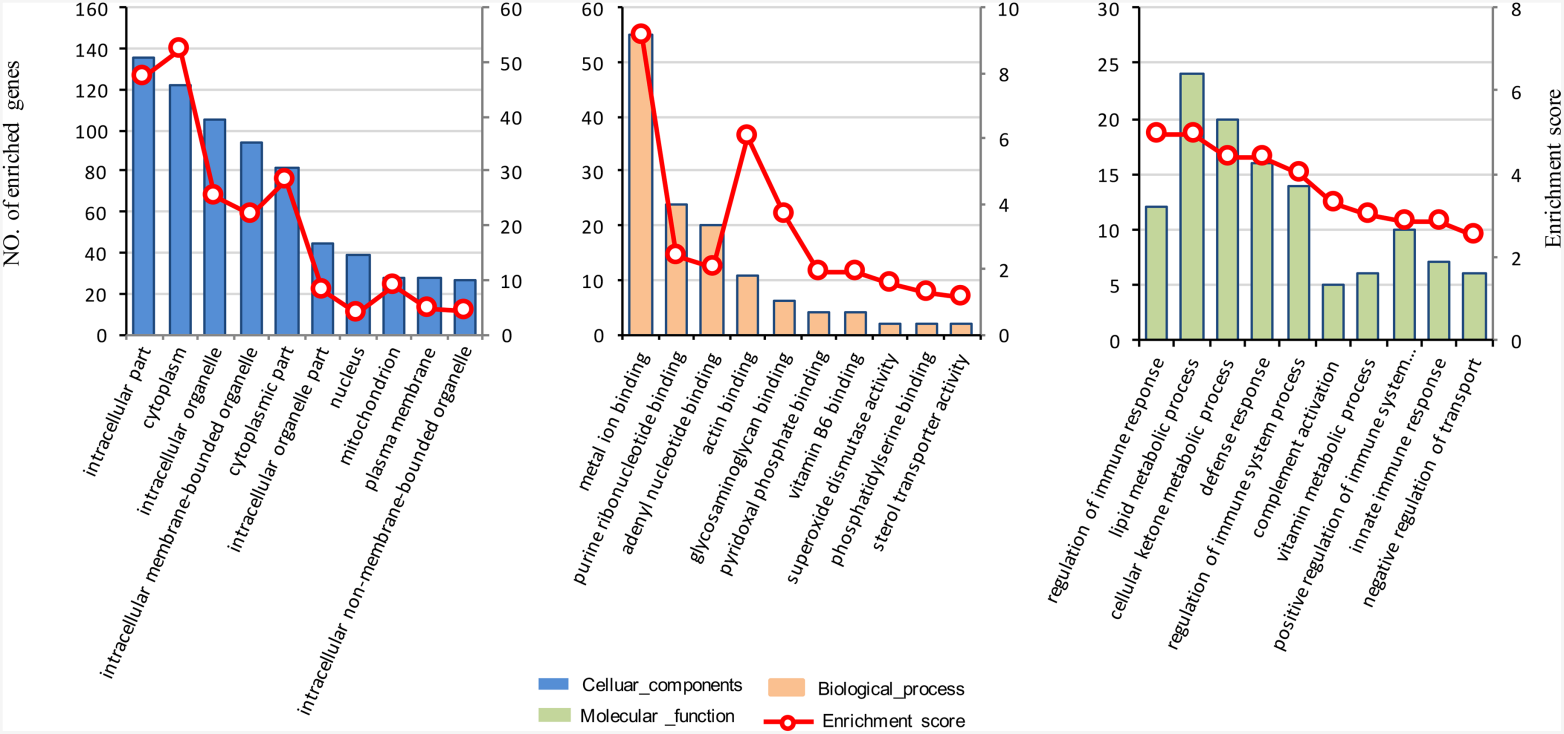
GO term of differential proteins for biological process, cellular components and molecular functions.

The DEPs were categorized according to their cellular components (CCs), molecular functions (MFs) and biological processes (BPs) (Fig 3). The cellular component annotation revealed that the significantly changed proteins were involved in intracellular part (GO:0044424), cytoplasm (GO:0005737), intracellular organelle (GO:0043229) and intracellular membrane-bounded organelle (GO:0043231). Molecular functions of the annotated proteins were primarily associated with three GO terms. The term of binding (GO: 0005488) contained several subterms including metal ion binding (GO:0046872), purine ribonucleotide binding (GO:0032555), actin binding (GO:0003779), and vitamin B6 binding (GO:0070279). The term of superoxide dismutase activity (GO:0004784) encompassed superoxide oxidoreductase activity of the catalysis of the reaction of superoxide to hydrogen peroxide. Sterol transporter activity (GO:0015248), a sub-term of transporter activity (GO: 0005215), involves small-molecule carriers or transporters that allow the intracellular movement or intercellular transport of macromolecules, small molecules and ions. The biological process annotation revealed that the significantly changed proteins were involved in four classes of GO terms. The immune system process (GO:0002376), which included regulation of immune response (GO:0006955), regulation of immune system process (GO:0002682), and innate immune response (GO:0045087), is involved in the development or functioning of the immune system. The metabolic processes were chemical reactions and pathways including anabolism and catabolism, small molecules transformation, and DNA repair and replication. The defense response (GO:0006952) and regulation of response to stimulus (GO:0048583) were subterms of response to stress (GO:0006950) that reflect a change in state or activity as a result of a disturbance in organismal or cellular homeostasis. The term of negative regulation of transport (GO:0051051) prevents or reduces the extent of the directed movement of substances into a cell or between cells.

**Fig 4:**
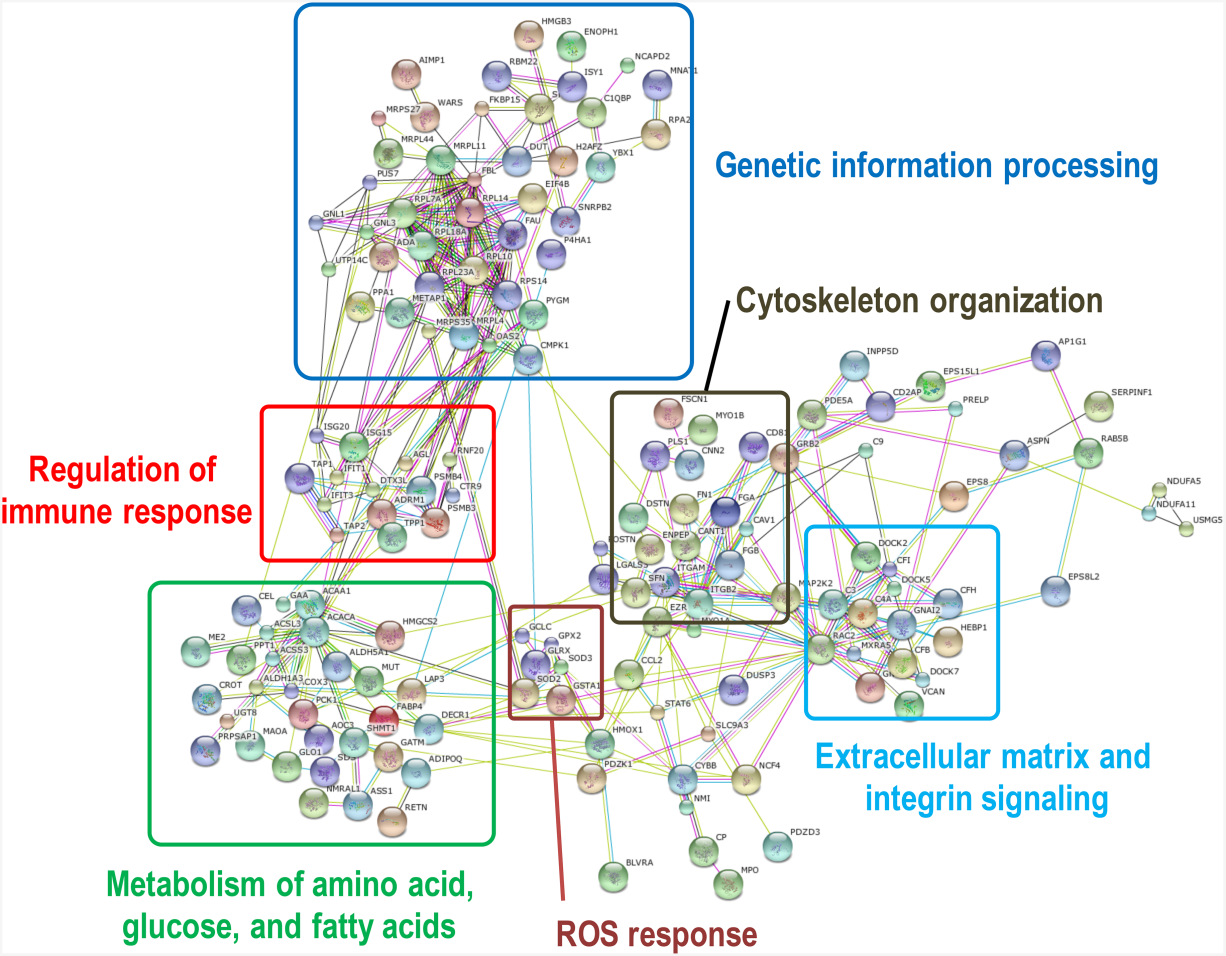
Protein-protein interaction network from a web-based search of the STRING database.

Additionally, KEGG pathway enrichment and protein-protein interaction network (PPI) analyses were used to determine the overrepresented biological events and to provide a primary overview of the jejunum proteome influenced by weaning stress. Pathway enrichment analysis showed that 340 differentially expressed proteins participated in 215 pathways. As shown in Table 1, some pathways were associated with metabolism (such as “Metabolic pathways” and “Pyruvate and Arachidonic acid metabolism”, among others) and immune regulation (such as “Complement and coagulation cascades”, “Phagosome” and “Antigen processing and presentation”, among others.). With DEPs as seed nodes, a protein-protein interaction network was constructed using the STRING Database version 9.0 (Fig 4; S4 Fig). The results showed that DEPs were primarily enriched for the “regulation of immune response”, “metabolism of amino acids, glucose, and fatty acids”, “genetic information processing”, “cytoskeleton organization”, and “extracellular matrix and integrin signaling”.

**Table 1:** Pathway enrichment analysis of differentially expressed proteins (DEPs).

Pathway name	Proteins number	P value	Pathway ID
**KEGG pathways enriched with Up-regulated proteins**			
Ribosome	11	2.08E-05	ko03010
Metabolism of xenobiotics by cytochrome P450	6	0.001832	ko00980
Glutathione metabolism	6	0.001832	ko00480
Drug metabolism—cytochrome P450	5	0.00353	ko00982
Folate biosynthesis	2	0.010485	ko00790
Linoleic acid metabolism	3	0.018846	ko00591
Primary immunodeficiency	3	0.027021	ko05340
Mineral absorption	3	0.033363	ko04978
Arachidonic acid metabolism	4	0.036653	ko00590
Herpes simplex infection	7	0.049862	ko05168
**KEGG pathways enriched with Down-regulated proteins**			
Complement and coagulation cascades	17	2.08E-10	ko04610
Staphylococcus aureus infection	13	1.99E-06	ko05150
Phagosome	20	9.94E-06	ko04145
PPAR signaling pathway	10	5.77E-05	ko03320
Pertussis	9	0.001096	ko05133
Glycine, serine and threonine metabolism	6	0.001328	ko00260
Tuberculosis	14	0.001605	ko05152
Phenylalanine metabolism	4	0.002865	ko00360
Proximal tubule bicarbonate reclamation	5	0.002929	ko04964
Bile secretion	7	0.002938	ko04976
Leishmaniasis	8	0.006186	ko05140
Lysosome	10	0.006993	ko04142
Cell adhesion molecules (CAMs)	9	0.007432	ko04514
Pyruvate metabolism	6	0.008534	ko00620
Malaria	4	0.012313	ko05144
Taurine and hypotaurine metabolism	2	0.013531	ko00430
Starch and sucrose metabolism	5	0.018679	ko00500
Type I diabetes mellitus	5	0.02116	ko04940
Natural killer cell mediated cytotoxicity	8	0.022149	ko04650
Porphyrin and chlorophyll metabolism	4	0.024129	ko00860
Tyrosine metabolism	4	0.02787	ko00350
Biosynthesis of unsaturated fatty acids	3	0.028574	ko01040
Legionellosis	6	0.028817	ko05134
Systemic lupus erythematosus	8	0.029734	ko05322
Allograft rejection	5	0.033185	ko05330
Mineral absorption	4	0.036337	ko04978
Autoimmune thyroid disease	5	0.040519	ko05320
beta-Alanine metabolism	4	0.041067	ko00410

The MGI database showed many phenotypes associated with intestinal morphology and functions. We observed many proteins involved in intestinal morphology, such as Keratin8 (Krt8) and Plastin1 (Pis1), which were annotated with terms such as “abnormal intestinal epithelium morphology” and “decreased small intestinal microvillus size”. We also observed many proteins related to intestinal absorption, such as Carboxyl ester lipase (Cel) and Transferrin receptor (Tfrc), which were annotated with terms such as “abnormal intestinal lipid absorption” and “abnormal intestinal mineral absorption”. Furthermore, many differentially expressed proteins, including Lectin galactose binding soluble 3 (Lgals3), Signal transducer and activator of transcription 6 (Stat6) and Complement component 3 (C3), were associated with phenotypes such as “small intestinal inflammation”, “decreased IgA level” and “abnormal immune system physiology” (S4 Table).

### Validation of gene expression by qRT-PCR

**Fig 5:**
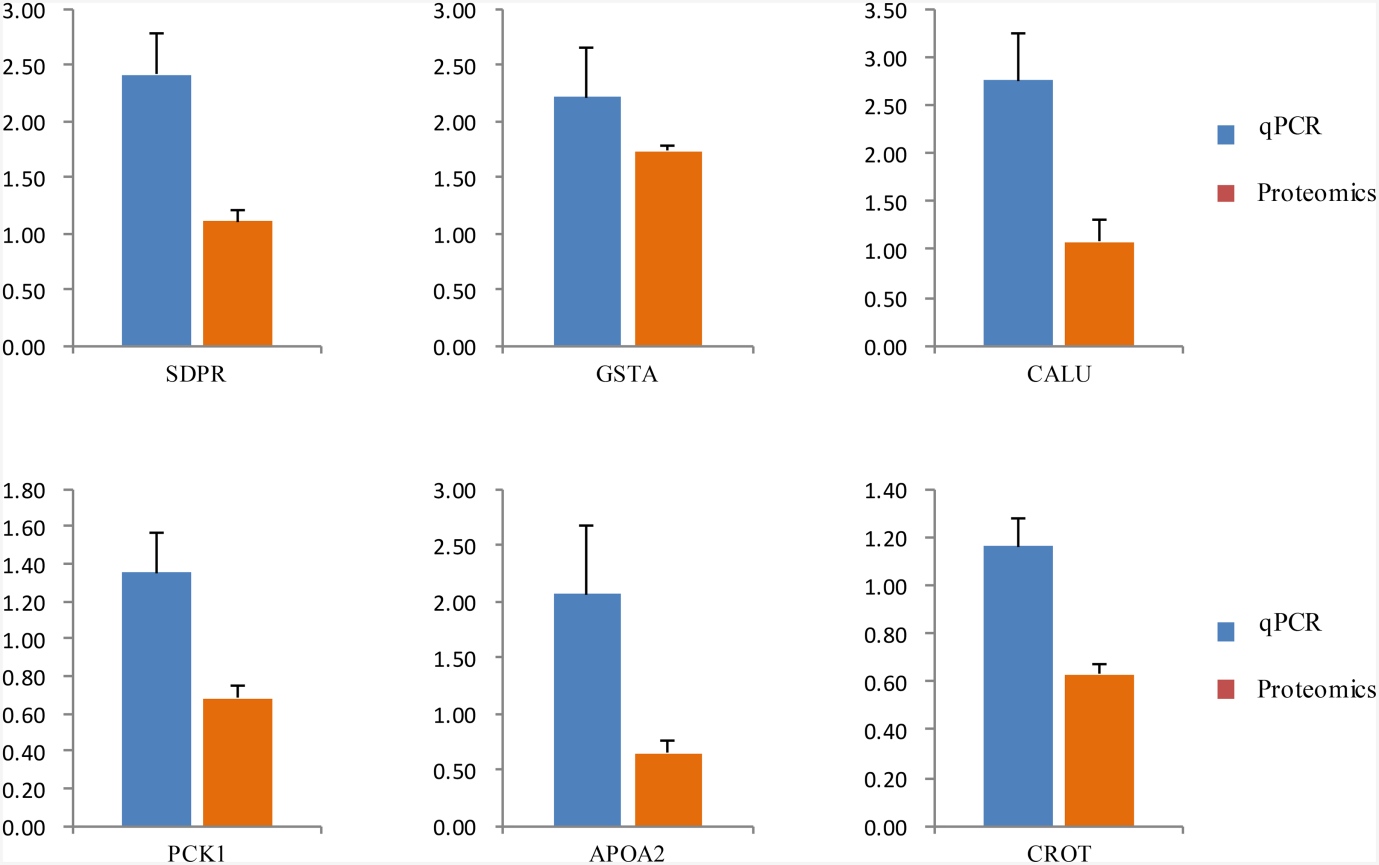
Comparison of mRNA expression ratios between AR and ER groups for selected genes.

Changes in protein expression may be due to changes in the mRNA level. In this study, to investigate whether the changes observed in protein expression were the result of transcriptional regulation, we used qRT-PCR to determine mRNA levels of six selected proteins that were related to lipid metabolic processes, defense response, regulation of immune response and intestinal epithelium morphology. As shown in Fig 5, the changes in mRNA expression of the genes were generally correlated with the corresponding changes in protein expression detected by the iTRAQ approach.

## Discussion

The gastrointestinal system has multiple functions, such as to digest and absorb nutrients and electrolytes; to maintain a balance of bodily fluids, and secrete digestive enzymes, mucin, immunoglobulins, and a variety of other components; and to serve as a barrier for the host against harmful pathogens and antigens. Any damage to the intestine can trigger severe health issues in the body. Separation from the dam at weaning is an important stressor of lambs that can contribute to intestinal and immune system dysfunctions. The focus of previous studies was primarily on technological improvements in housing, nutrition, health, and management, which have been used to minimize the adverse effects of weaning stress [19–22]. However, much less is understood regarding the molecular mechanisms in the intestine during weaning stress. In this study, iTRAQ technology combined with LC-ESI-MS/MS was applied to investigate DEPs in the intestine of twin lambs during weaning stress. This combination of approaches led to accurate identification of peptides and precise quantification of iTRAQ labels with a wide scan range. Subsequently, different methods of functional clustering methods for the proteins of interest were focused to obtain consistent results for the metabolism of amino acids, glucose, and fatty acids, the immune response, the cytoskeleton organization, and the extracellular matrix and integrin signaling for weaning stress.

### Intestinal structure and nutrient absorption and transportation

The primary functions of the intestine are the absorption of nutrients and as being a physical barrier [23]. The small intestine is the site in which most nutrients from ingested feed are absorbed. Villi are small, finger-like projections that protrude from the epithelial lining of the intestinal wall that increase the internal surface area of the intestinal walls, forming a greater surface area for nutrient absorption (including monosaccharides and amino acids) through either diffusion or active transport [24]. Many studies have confirmed that weaning induces both acute and long-lasting structural and functional changes in the intestine, such as shortening of the villi (villous atrophy) and increasing crypt depth (crypt elongation) after weaning [5, 11].

In this study, intestinal histological sections showed that the villi morphology of the intestine was affected at this critical stage. The proteomic analysis identified many differentially expressed proteins that were considerably enriched in GO categories and KEGG pathways associated with the cytoskeleton and metabolic processes. The GO terms of actin binding (GO: 0003779) interact selectively and non-covalently with monomeric or multimeric forms of actin, including actin filaments, which play a significant role in the morphological maintenance of villi [25].

### Alterations in metabolic processes

In our research, the most represented GO terms were lipid metabolic processes, cellular amino acids and derivative metabolic processes and carbohydrate metabolic processes. The significantly enriched metabolism-associated pathways included glycine, serine and threonine metabolism; the PPAR signaling pathway; phenylalanine metabolism; pyruvate metabolism; taurine and hypotaurine metabolism; starch and sucrose metabolism; tyrosine metabolism; and beta-alanine metabolism. Of note these pathways were down regulated after weaning, which suggested that these metabolic processes were most likely disturbed by weaning stress.

Nutrient absorption in the intestine occurs through either diffusion or active transport. The proteomics data in our research showed that the DEPs enriched in GO terms were down regulated during weaning stress; these proteins, such as those involved in transporter activity, are involved in the directed movement of substances (such as macromolecules, small molecules, ions) into, out of or within a cell or between cells. Binding was the primary function under the category of molecular functions of the GO analysis, and many down-regulated proteins were involved in this process. Moreover, 13 down-regulated proteins were enriched in the pathway of cell adhesion molecules (CAMs) and focal adhesion, which are associated with communication between cells. The down-regulated proteins related to the function of transport, binding and cell junctions might be part of the explanation for the decline in absorption of nutrients during weaning stress.

### Disturbance of immune regulation processes in the intestine

In addition to the compromised digestive and absorptive capacities, consequences associated with weaning also induce a deleterious effect on intestinal barrier functions [11, 26, 27]. The epithelial layer of the intestinal lumen serves as the first line of defense for protecting livestock from various harmful microorganisms, toxins, or antigens. When the intestinal barrier is disrupted, the increase in permeability allows toxins, bacteria, or feed-associated antigens to cross the epithelium resulting in inflammation, mal-absorption, diarrhea, and reduced growth and production. Tight junctions are one mode of cell-to-cell adhesion in epithelial and endothelial cellular sheets that act as a primary barrier to the diffusion of solutes through the intercellular space, in addition to recruiting various cytoskeletal and signaling molecules at their cytoplasmic surfaces [28, 29]. Previous research shows that dysfunctional tight junctions lead to increased intestinal permeability [29]. In this study, 3 down-regulated proteins (Lasp1, Gnai2 and Ppp2ca) were enriched in the pathway of tight junctions.

In addition to the increase in permeability associated with a dysfunctional intestinal barrier, weaning induces a breakdown in mucosal inflammation. The primary function of the mucosal immune system is defense against potential pathogens that may enter across the vulnerable surface epithelia. During the postnatal period, piglets face many new antigens, and at weaning, a second wave of nutritional antigens enters the intestinal tract.

Host defense against microbial pathogens requires appropriate coordination of multiple signaling pathways. In this study, we found that differentially expressed proteins enriched in KEGG pathways included complement and coagulation cascades, phagosomes, cell adhesion molecules (CAMs), and the MAPK signaling pathway. Several differentially expressed proteins in the intestine, such as fibrinogen proteins (FGAs) and complement proteins (C3, C7 and C9), participate in many pathways encompassing the complement and coagulation cascades and the MAPK signaling pathway. The complement and coagulation cascades are connected to the immune system [30]. The complement system plays a fundamental role in innate immunity, in addition to enhancing adaptive immune responses, and therefore is a primary line of defense against infection [31]. MAPK plays a key role in activating host innate immune responses and is a frequent target of pathogenic effectors in animal systems [32]. Phagocytosis is a central mechanism in tissue remodeling, inflammation, and defense against infectious agents. The fusion of phagosomes and lysosomes releases toxic products that kill most bacteria and degrade them into fragments. Cell adhesion molecules are (glyco) proteins expressed on the cell surface and play a critical role in a wide array of biologic processes, including hemostasis, the immune response and inflammation. These down-regulated proteins involved in the regulation of immune processes might be the ultimate cause for vulnerability and high mortality rates in weaning lambs.

## Conclusions

In this study, iTRAQ technology was used for the first time to demonstrate proteome spectrum changes in the intestines of lambs during weaning stress. Moreover, twin lambs were used in the study to minimize the genetic differences between the two groups. It was concluded that weaning stress results in changes of the intestinal morphology and functions which might be attributed to DEPs. The differentially expressed proteins enriched in gene ontology categories and KEGG pathways associated with the cytoskeleton likely played a significant role in the morphological maintenance of villi. The down-regulated proteins related to the function of transport, binding and cell junctions might explain the decrease in absorption of nutrients and with these same proteins involved in immunoregulation might be the ultimate reason for the vulnerability and high mortality rates in weaning lambs. These advanced proteome data have expanded our knowledge significantly and have provided new insights into the effects of weaning stress on the intestine.

## Supporting information

S1 Fig Repeatability analysis of biological replicates. (DOCX)

S2 Fig Cluster of orthologous group (COG) classification of identified proteins. (DOCX)

S3 Fig Generation of iTRAQ data. (DOCX)

S4 Fig Protein-protein interaction network from a web-based search of the STRING Database. (PPTX)

S1 Table The qPCR primers used for verification of the differentially expressed genes of the intestine. (XLSX)

S2 Table The identified proteins of the intestine stress. (XLSX)

S3 Table The different expressed proteins of the intestine. (XLSX)

S4 Table MGI phenotype annotations using different expressed proteins. (XLSX)

## References

[1] Flower FC, Weary DM. Effects of early separation on the dairy cow and calf: 2. Separation at 1 day and 2 weeks after birth. Appl Anim Behav Sci. 2001, 70(4): 275–284. 11179551 10.1016/s0168-1591(00)00164-7

[2] Gu X, She DLR. Effect of weaning on small intestinal structure and function in the piglet. Arch Anim Nutr. 2002, 56(4): 275–286. 12462912 10.1080/00039420214345

[3] Campbell JM, Crenshaw JD, Polo J. The biological stress of early weaned piglets. J Anim Sci Biotechnol. 2013, 4(1): 19. 10.1186/2049-1891-4-19 23631414 PMC3651348

[4] Weary DM, Jasper J, Hötzel MJ. Understanding weaning distress. Appl Anim Behav Sci. 2008, 110(1–2): 24–41. 10.1016/j.applanim.2007.03.025

[5] Pluske JR, Hampson DJ, Williams IH. Factors influencing the structure and function of the small intestine in the weaned pig: a review. Livestock Production Science. 1997, 51(97): 215–236. 10.1016/S0301-6226(97)00057-2

[6] Bomba L, Minuti A, Moisa SJ, Trevisi E, Eufemi E, Lizier M, et al Gut response induced by weaning in piglet features marked changes in immune and inflammatory response. Funct Integr Genomics.2014,14(4):657–671. 10.1007/s10142-014-0396-x 25199657

[7] Pascual-Alonso M, MDLL Gc, Aguayo-Ulloa L, Ezquerro L, Villarroel M, Marín RH, et al Effect of postweaning handling strategies on welfare and productive traits in lambs. J Appl Anim Wel Sci. 2015, 18(1): 42–56. 10.1080/10888705.2014.941107 25105466

[8] Stokes CR, Bailey M, Haverson K, Harris C, Jones P, Inman C, et al Postnatal development of intestinal immune system in piglets: implications for the process of weaning. Animal Research. 2004, 53(4): 325–334. 10.1051/animres:2004020

[9] Bailey M, Haverson K, Inman C, Harris C, Jones P, Corfield G, et al The development of the mucosal immune system pre- and post-weaning: balancing regulatory and effector function. P Nutr Soc. 2005, 64(04): 451–457. 10.1079/PNS2005452 16313686

[10] Junjun W, Lixiang C, Defa L, Yulong Y, Xiaoqiu W, Peng L, et al Intrauterine growth restriction affects the proteomes of the small intestine, liver, and skeletal muscle in newborn pigs. J Nutr. 2008, 138(1): 60–66. 10.1093/jn/138.1.60 18156405

[11] Ga Lle B, Vincent P, Isabelle LHR, Jean Paul L, Bernard S. Weaning induces both transient and long-lasting modifications of absorptive, secretory, and barrier properties of piglet intestine. J Nutr. 2004, 134(9): 2256–2262. 10.1093/jn/134.9.2256 15333713

[12] Aw TY. Intestinal glutathione: determinant of mucosal peroxide transport, metabolism, and oxidative susceptibility. Toxicology & Applied Pharmacology. 2005, 204(3): 320–328. 10.1016/j.taap.2004.11.016 15845421

[13] Tao X, Xu Z, Men X. Transient changes of enzyme activities and expression of stress proteins in the small intestine of piglets after weaning. Arch Anim Nutr. 2015, 69(3): 201–211. 10.1080/1745039X.2015.1034828 25908169

[14] Meier RE, Steuerwald M. The role of nutrition in diarrhoea syndromes. Current Opinion in Clinical Nutrition & Metabolic Care. 2003, 6(5): 563–567 12913674 10.1097/00075197-200309000-00010

[15] Kong XF, Wu GY, Liao YP, Hou ZP, Liu HJ, Yin FG, et al Effects of Chinese herbal ultra-fine powder as a dietary additive on growth performance, serum metabolites and intestinal health in early-weaned piglets. Livest Sci. 2007, 108(1): 272–275. 10.1016/j.livsci.2007.01.079

[16] Yue GH. Reproductive characteristics of Chinese Hu sheep. Anim Reprod Sci. 1996, 44(4): 223–230. 10.1016/0378-4320(96)01562-X

[17] Hu PF, Li XC, Liu HK, Guan WJ, Ma YH. Construction and characterization of a cDNA expression library from the endangered Hu sheep. Genet Mol Res. 2014, 13(4): 9019–9023. 10.4238/2014.October.31.16 25366792

[18] Eckersall PD, de Almeida AM, Miller I. Proteomics, a new tool for farm animal science. J Proteomics. 2012, 75(14): 4187–4189. 10.1016/j.jprot.2012.05.014 22634566

[19] Napolitano F, De Rosa G, Sevi A. Welfare implications of artificial rearing and early weaning in sheep. Appl Anim Behav Sci. 2008, 110(1–2): 58–72. 10.1016/j.applanim.2007.03.020

[20] Johnsen JF, Beaver A, Mejdell CM, Rushen J, de Passille AM, Weary DM. Providing supplementary milk to suckling dairy calves improves performance at separation and weaning. J Dairy Sci. 2015, 98(7): 4800–4810. 10.3168/jds.2014-9128 25912862

[21] Orgeur P, Bernard S, Naciri M, Nowak R, Schaal B, Lévy F. Psychobiological consequences of two different weaning methods in sheep. Reproduction Nutrition Development. 1999, 39(2): 231–244. 10327451 10.1051/rnd:19990208

[22] Awad WA, Ghareeb K, Paßlack N, Zentek J. Dietary inulin alters the intestinal absorptive and barrier function of piglet intestine after weaning. Res Vet Sci. 2013, 95(1): 249–254. 10.1016/j.rvsc.2013.02.009 23523472

[23] Rebeca S, Abreu MT. Innate immunity in the small intestine. Curr Opin Gastroen. 2012, 27(2): 124–129. 10.1097/MOG.0b013e3283506559 22241076 PMC3502878

[24] Vente-Spreeuwenberg MAM, Verdonk JMAJ, Bakker GCM, Beynen AC, Verstegen MWA. Effect of dietary protein source on feed intake and small intestinal morphology in newly weaned piglets. Livestock Production Science. 2004, 86(03): 169–177.

[25] Saotome I, Curto M, Mcclatchey AI. Ezrin is essential for epithelial organization and villus morphogenesis in the developing intestine. Dev Cell. 2004, 6(6): 855–864. 10.1016/j.devcel.2004.05.007 15177033

[26] Adam J M, Carin Vander K, Kathleen A R, Jenna G W, Dianne L, Vanessa L C, et al Stress signaling pathways activated by weaning mediate intestinal dysfunction in the pig. American Journal of Physiology Gastrointestinal & Liver Physiology. 2008, 292(1): G173–G181. 10.1152/ajpgi.00197.2006 16901995

[27] Spreeuwenberg MA, Verdonk JM, Gaskins HR, Verstegen MW. Small intestine epithelial barrier function is compromised in pigs with low feed intake at weaning. J Nutr. 2001, 131(5): 1520–1527. 10.1093/jn/131.5.1520 11340110

[28] Luca M, Venanzio V, La Torre Giuseppe, Massimo M, Giovanni C, Riccardo R, et al Increased intestinal permeability and tight junction alterations in nonalcoholic fatty liver disease. Hepatology. 2009, 49(6): 1877–1887. 10.1002/hep.22848 19291785

[29] Terry S, Nie M, Matter K, Balda MS. Rho signaling and tight junction functions. Physiology. 2010, 25(1): 16–26. 10.1152/physiol.00034.2009 20134025

[30] Oikonomopoulou Katerina Ricklin, Ward Daniel, Lambris Peter A, John D. Interactions between coagulation and complement—Their role in inflammation Seminars in Immunopathology. 2011,151–165. 10.1007/s00281-011-0280-x 21811895 PMC3372068

[31] Carroll MC. Complement and humoral immunity. Vaccine. 2008, 26(30): I28–I33. 19388161 10.1016/j.vaccine.2008.11.022 PMC4018718

[32] Y JA, D A. MAPping innate immunity. P NatI Acad Sci Usa. 2004, 101(35): 12781–12782. 10.1073/pnas.0404890101 15328410 PMC516471

