# Supplementary figures and images for: iTRAQ-based quantitative proteomic analysis of alterations in the intestine of Hu sheep under weaning stress
Source: PLoS One. 2018 Jul 19;13(7):e0200680. doi: 10.1371/journal.pone.0200680 (PMC6053177; doi:10.1371/journal.pone.0200680)

Fig S1. Repeatability analysis of biological replicates


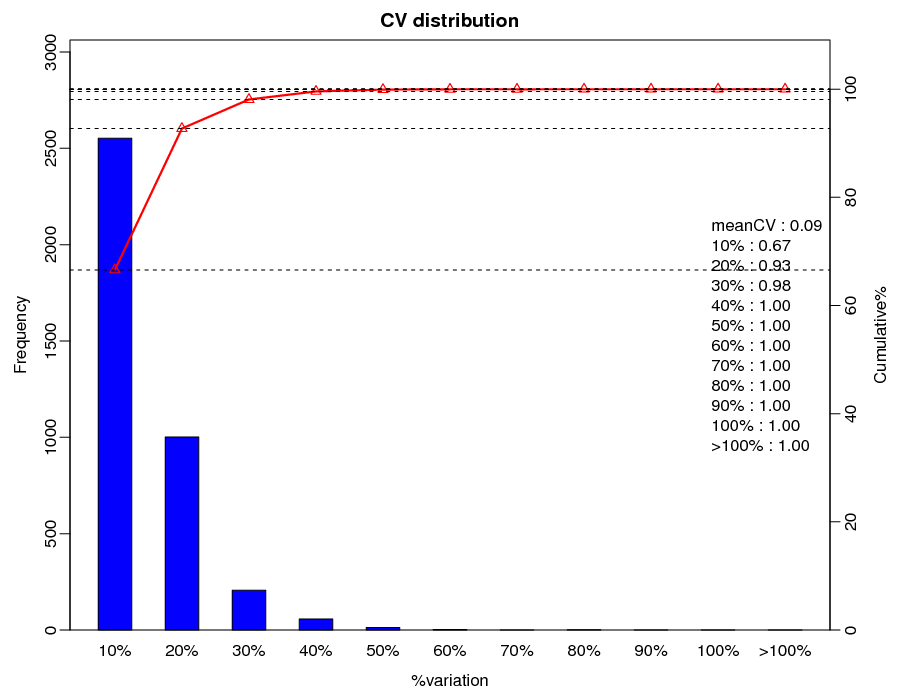

Supplement: S1 Fig — (DOCX) [file pone.0200680.s001.docx]

Fig S2. Cluster of orthologous groups (COG) classification of identified proteins


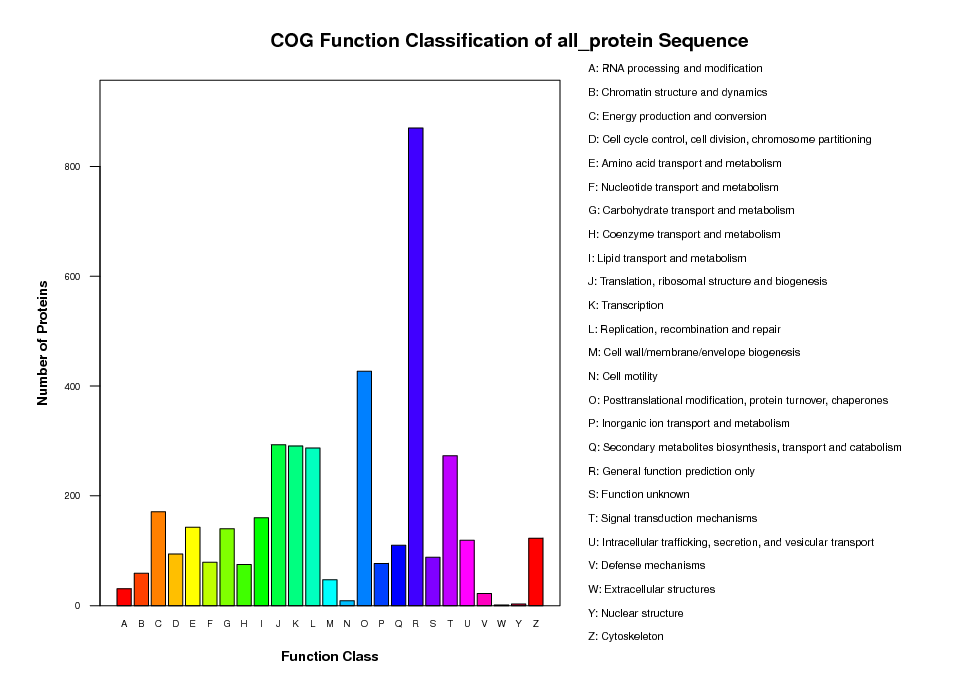

Supplement: S2 Fig — (DOCX) [file pone.0200680.s002.docx]
