# Supplementary material for: iTRAQ-based quantitative proteomic analysis of alterations in the intestine of Hu sheep under weaning stress
Source: PLoS One. 2018 Jul 19;13(7):e0200680. doi: 10.1371/journal.pone.0200680 (PMC6053177; doi:10.1371/journal.pone.0200680)
Supplement: S3 Fig — (DOCX) [file pone.0200680.s003.docx]

**Figure S3. Generation of iTRAQ data.** (A) Mascot identification statistics. “Total Spectra” indicates the number of all spectra spectrograms detected by iTRAQ. “Spectra” indicates the number of identified spectra spectrograms. “Unique Spectra” indicates the number of spectra spectrograms matching unique peptide sequences. “Peptide” indicates the number of identified peptide sequences. “Unique Peptide” indicates the number of unique peptide sequences in the identified proteins. “Protein” indicates the number of identified proteins. (B) Mass distribution of the identified proteins. The number of proteins distributed in different ranges of molecular mass are shown in the graph. (C) Distribution of protein’s sequence coverage. The number of proteins distributed in different ranges of peptide sequence coverage is shown in the graph. (D) Distribution of the number of peptides for the identified proteins. The identified proteins are grouped based on the number of matched peptides. The number of proteins in each group are counted in the graph.
