# Supplementary material for: iTRAQ-based quantitative proteomic analysis of alterations in the intestine of Hu sheep under weaning stress
Source: PLoS One. 2018 Jul 19;13(7):e0200680. doi: 10.1371/journal.pone.0200680 (PMC6053177; doi:10.1371/journal.pone.0200680)
Supplement: S4 Fig — (PPTX) [file pone.0200680.s004.pptx]

## Slide 1
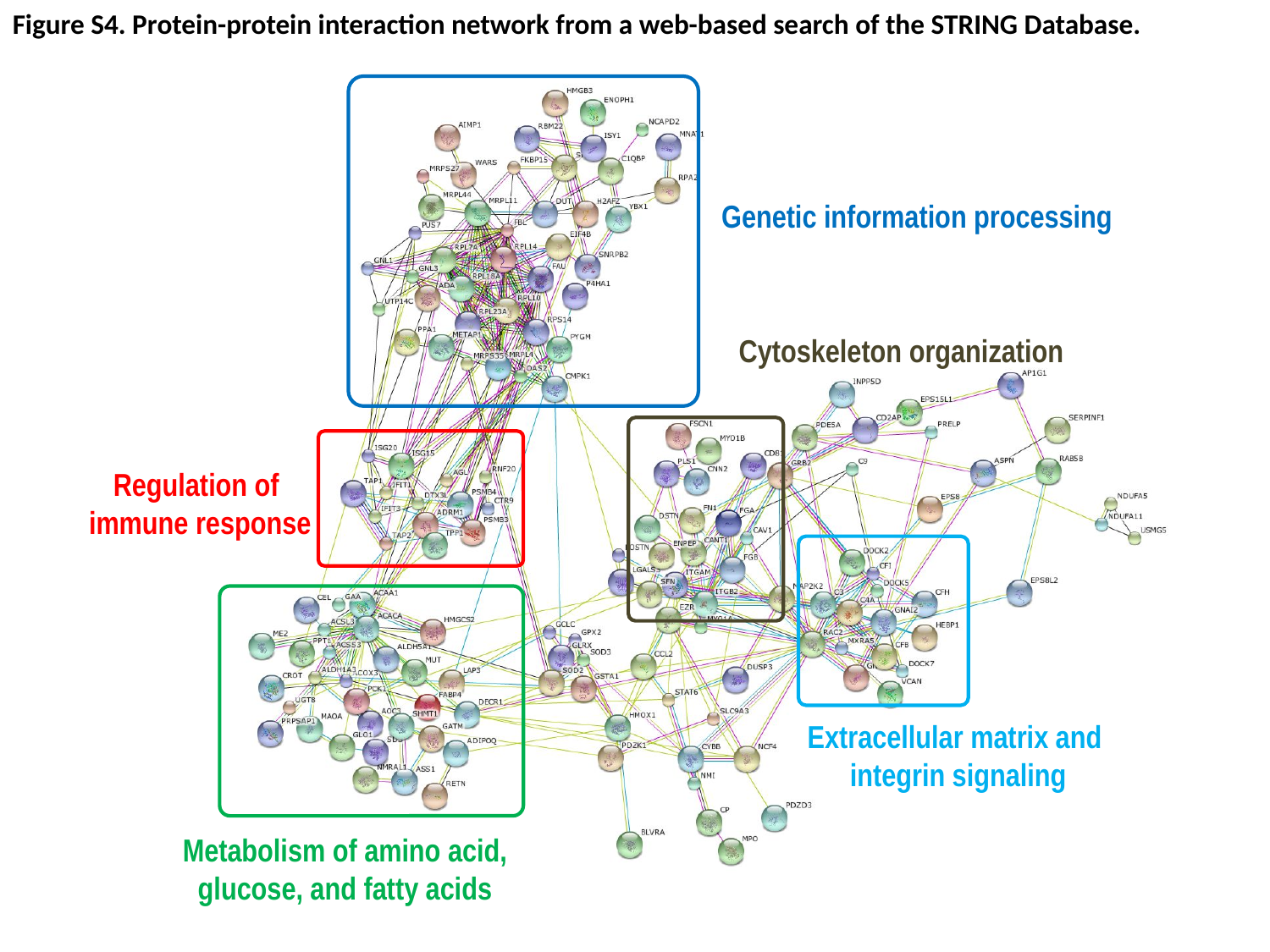

Figure S4. Protein-protein interaction network from a web-based search of the STRING Database.
Genetic information processing
Cytoskeleton organization
Regulation of
immune response
Extracellular matrix and
integrin signaling
Metabolism of amino acid,
glucose, and fatty acids
